# Scutellarin protects against doxorubicin-induced acute cardiotoxicity and regulates its accumulation in the heart

**DOI:** 10.1007/s12272-017-0907-0

**Published:** 2017-03-17

**Authors:** Xi-Peng Sun, Li-Li Wan, Quan-Jun Yang, Yan Huo, Yong-Long Han, Cheng Guo

**Affiliations:** Department of Pharmacy, Shanghai Jiao Tong University Affiliated Sixth People’s Hospital, No. 600, Road Yishan, Shanghai, 200233 People’s Republic of China

**Keywords:** Doxorubicin, Scutellarin, Cardiotoxicity, Oxidative stress, Tissue distribution

## Abstract

The clinical use of doxorubicin (DOX) is limited by its dose-dependent cardiotoxicity. The present study investigated the effects of scutellarin against DOX-induced cardiotoxicity in rats using pharmacodynamic and pharmacokinetic approaches. DOX (20 mg/kg) was injected intraperitoneally (i.p.) as a single dose, and scutellarin (5 mg/kg/day) was injected intravenously (i.v.) for 3 days. Rats treated with DOX showed acute cardiotoxicity as indicated by the elevated serum lactate dehydrogenase (LDH) activity (4057.8 ± 107.2 vs. 2032.7 ± 70.95), tissue malondialdehyde (MDA) level (2.083 ± 0.10 vs. 1.103 ± 0.09), cardiac troponin T (cTnT) concentration (0.1695 ± 0.0114 ng/mL), the decreased left ventricular ejection fraction (LVEF) (47.75 ± 15.79 vs. 78.72 ± 7.25) and left ventricular fractional shortening (LVFS) (20.66 ± 8.06 vs. 43.7 ± 6.76) compared with those of the control group. Cotreatment with scutellarin significantly decreased the LDH activity (2595.9 ± 72.73), MDA level (1.380 ± 0.06), cTnT concentration (0.0222 ± 0.0041 ng/m L), increased LVEF (76.70 ± 3.91) and LVFS (40.28 ± 3.68). Histopathological studies showed disruption of cardiac tissues in the DOX groups. Cotreatment with scutellarin reduced the damage to cardiac tissues. In the pharmacokinetic and tissue distribution study, scutellarin reduced the heart tissue exposure to DOX but did not change the AUC of plasma. These results suggest that scutellarin can protect against DOX-induced acute cardiotoxicity through its antioxidant activity and alterations of heart concentrations.

## Introduction

Doxorubicin (DOX) is an effective antineoplastic agent that is used to treat a wide spectrum of human malignancies and remains one of the most commonly prescribed drugs (Pautier et al. 2015; Tap et al. 2016). However, its clinical usage is limited by the development of severe dose-dependent acute and chronic cardiomyopathy (Manalo et al. 1975; Carvalho et al. 2014), which is usually refractory to common medications. Approximately 10% of patients treated with DOX will develop cardiomyopathy (Lipshultz et al. 2010).The pathogenic mechanisms responsible for DOX-induced cardiotoxicity have not been completely elucidated. It is widely accepted that DOX-induced oxidative stress plays an important role in the pathogenesis of DOX cardiotoxicity (Guenancia et al. 2015; Guo et al. 2016; Szwed et al. 2016). The quinone moiety of DOX participates in oxidation–reduction processes and promotes ROS generation through an enzymatic mechanism that utilizes the mitochondrial respiratory chain and non-enzymatic pathways that incorporate iron (Minotti et al. 2004). Furthermore, cardiomyocytes exhibit low levels of antioxidases, such as catalase and superoxide dismutase, and the antioxidant seledium-dependent-reduced GSH peroxidase-1 is readily inactivated after DOX exposure. It has been shown that DOX has a high affinity for cardiolipin and forms a very stable complex, resulting in DOX accumulation in the heart and aggravation of the heart burden (Moulin et al. 2015; Aryal and Rao 2016). DOX-induced cardiotoxicity could be attenuated by reducing heart exposure to DOX, such as with liposome-encapsulated DOX (Batist et al. 2001).

Because DOX is such an effective chemotherapeutic, a variety of approaches have been evaluated to reduce its cardiotoxicity, including DOX analogues, alternative drug-delivery methods, and an iron-chelating agent. One method of attenuating the risk of DOX-induced cardiotoxicity and exploiting the full therapeutic potential of DOX is a cardioprotective agent that does not affect its antitumor activity.

Several recent studies have proposed flavonoids, a group of potent cardioprotective agents, to protect against DOX-induced cardiotoxicity. Scutellarin (SCU, 4,5,6-trihydroxyflavone-7-glucuronide) is a natural flavonoid and the primary active ingredient of *Erigeron breviscapus* (vant.) Hand-Mazz, a Chinese herbal medicine. Its preparations for injection are widely used in the clinic for the treatment of cerebral insufficiency and peripheral circulation problems in China. Many studies have demonstrated that scutellarin is an effective radical scavenger in vitro and in vivo. Scutellarin could attenuate H_2_O_2_-induced oxidative stress and lipid peroxidation (Liu et al. 2003; Hong and Liu 2004). In addition, recent studies have suggested that scutellarin exerts a cardioprotective effect against infarction and ischemia–reperfusion injury (Lin et al. 2007).The radical scavenging and anti-oxidation properties of flavonoids might be beneficial to decrease DOX-induced cardiotoxicity. Furthermore, scutellarin was shown to be effective in the treatment of cardiovascular diseases, and it is commonly used for dilating blood vessels, improving coronary flow, and protecting cardiac microvascular endothelial cells (Liu et al. 2005; Chen et al. 2006; Tang et al. 2015). Based on the potential role of scutellarin in ameliorating oxidative stress and injury, this study aimed to investigate the beneficial effect of scutellarin against DOX-induced cardiotoxicity from a pharmacodynamics and pharmacokinetic perspective.

## Materials and methods

### Reagents

The reference standard of doxorubicin was purchased from the China Pharmaceutical Biological Products Analysis Institute (Shanghai, China). Doxorubicin hydrochloride was purchased as a powder for injection from Shenzhen Main Luck Pharmaceuticals Inc. (Shenzhen, China). Scutellarin (purity >98%) was purchased as a powder from Shanghai Winherb Medical Science Co. Ltd (Shanghai, China). MDA, LDH, and Coomassie brilliant blue kits were purchased from the Nanjing Jiancheng Bio-engineering Institute (Nanjing, China). All solvents used in the extraction and HPLC analyses of DOX were HPLC grade and were purchased from Tedia (Tedia, USA).

### Animals

All procedures involving animals and their care in this study were approved by the animal care committee of our institution in accordance with institutional requirements and Chinese government guidelines for animal experiments. Male Sprague–Dawley rats (200–250 g weight) were purchased from Sino-British Sippr/BK Lab Animal Ltd. (Shanghai, China). The rats were maintained in pathogen-free conditions with a constant temperature (24 ± 2 °C) and humidity (relative humidity of 55 ± 15%) and a 12-h light/dark cycle. They had free access to water and identical standard food (when permitted) for all subjects, and the experiments were started after 1 week of acclimation.

### Experimental protocol

#### Experiment 1

Thirty-two rats, fasted overnight before the experiments, were randomly divided into four groups (*n* = 8 in each group) and treated as follows: the control group received a single i.p. injection of saline on day 1; the SCU group received 5 mg/kg/day i.v. scutellarin via the tail vein on day 1, day 2 and day 3; the DOX group received a single i.p. dose of 20 mg/kg DOX on day 1, followed immediately by i.v. saline via the tail vein on day 1, day 2 and day 3; the SCU + DOX group received a single i.p. dose of 20 mg/kg DOX on day 1, followed immediately by i.v. 5 mg/kg scutellarin via the tail vein on day 1, day 2 and day 3.The dose and injection regimens for DOX were based on published previously reports (Riad et al. 2009). On the 4th day, the animals were anesthetized by amobarbital. Then, transthoracic echocardiography was performed on anesthetized animals. Blood samples were collected from the abdominal aorta, and the serum was separated. The hearts were rapidly excised after sacrifice, thoroughly washed with cold saline, and then cut into two parts. The left ventricular wall was fixed in 10% formalin for histological examination. The other part of the heart tissue was stored at −20 °C until analysis in the lipid peroxide assay.

#### Experiment 2

Sixteen rats, fasted overnight before the experiments, were randomly assigned to the DOX group (*n* = 8) or DOX + SCU group (*n* = 8). The DOX group was given an i.v. single dose of 5.0 mg/kg DOX via the tail vein. The DOX + SCU group was given an i.v. single dose of 5.0 mg/kg DOX and a single dose of 5.0 mg/kg scutellarin 5 min after the bolus of DOX via a tail vein. The right external jugular vein was cannulated for sample collection, and 0.5 mL blood samples were collected into heparinized tubes from the jugular vein and withdrawn at 5 min, 0.5, 1, 2, 3, 4, 6, 8, 10, 24, 32, 48, and 72 h after administration. The plasma was isolated by centrifugation and stored at −20 °C until analysis. At 72 h after the blood collection, the rats were sacrificed, and the hearts were rapidly excised. The hearts were thoroughly washed with cold saline and stored at −20 °C until analysis.

#### Experiment 3

Sixty rats, fasted overnight before the experiments, were randomly assigned to the DOX group (*n* = 30) or DOX + SCU group (*n* = 30). The DOX group was given an i.v. single dose of 5.0 mg/kg DOX via the tail vein. The DOX + SCU group was given an i.v. single dose of 5.0 mg/kg DOX and a single dose of 5.0 mg/kg scutellarin 5 min after the bolus of DOX via a tail vein. Six rats from the DOX group and DOX + SCU group were euthanized at 1, 4, 24, 48, and 72 h after DOX administration. Samples of the heart, liver, spleen, lung, kidney, gut, and muscle were excised, and they were then washed thoroughly with cold saline and stored at −20 °C until analysis.

### Assay of lipid peroxide level

The concentration of free MDA in the heart, a product formed due to cell membrane peroxidation, was assessed using an MDA kit according to the manufacturer’s instructions. In brief, the assay was based on the reaction of MDA with thiobarbituric acid (TBA), forming stable thiobarbituric acid-reactive substances (TBARS) that absorb at 532 nm. The lipid peroxide level was expressed as nmol of MDA per mg protein.

### Assay of lactate dehydrogenase (LDH) activity

The LDH activity in serum was estimated using a LDH kit according to the manufacturer’s instructions by a UV–Vis spectrophotometer.

### Determination of protein content

The protein content of serum and cardiac tissue homogenates was determined by a Coomassie brilliant blue kit according to the manufacturer’s instructions with a UV–Vis spectrophotometer.

### Concentration of cardiac troponin T (cTnT) in blood serum

The concentrations of cTnT in the serum were determined by a chemiluminescence immunoassay (Roche Elecsys 2010). The assay was performed in each group, and the results are expressed as ng/ml.

### Echocardiographic assessment of cardiac physiological alterations

Transthoracic echocardiography (Siemens Sequoir 512) was performed on anesthetized animals with a linear array 8–12 MHz transducer on the 4th day. M-mold images were acquired and used for the calculation of LV function using calculation procedures. Left ventricular ejection fraction (LVEF) is calculated from end-diastolic volume (EDV) and end-systolic volume (ESV), using the following formula: LVEF = (EDV − ESV)/EDV. EDV and ESV were estimated by the Teichholtz method. Left ventricular fractional shortening (LVFS) is calculated from the difference of M-mold derived end-diastolic diameter and end-systolic diameter divided by end-diastolic diameter.

### Histopathological studies

The left ventricular walls of heart tissues were fixed in 10% formalin, routinely processed, and embedded in paraffin. Sections were cut at 3–5 μm thickness and stained with hematoxylin and eosin (H&E) for histological examination. The sections were then viewed under a light microscope. A histomorphological evaluation of all the heart sections was carried out by a pathologist who was blinded to the treatment groups.

### High performance liquid chromatography (HPLC) analysis of DOX in plasma and tissues

The DOX concentration in plasma and tissues was analyzed using HPLC with fluorometric detection. The tissue samples were homogenized in ten times the volume of ice-cold saline. Briefly, 20 μL internal standard (20 μg/mL pirarubicin) and 200 μL of alcohol were added to a 200μL aliquot of plasma or homogenate, followed by vortexing for 0.5 min. This mixture was extracted with 600 μL of dichloromethane. The organic phase (600 μL) was separated, evaporated to dryness, reconstituted with methanol, and injected into the HPLC. The Agilent 1100 HPLC Series system was equipped with ChemStation software, a 1100-well plate autosampler, online degasser, and a fluorescence detector. Separation was achieved on a C18 reverse-phase column at 30 °C using acetonitrile and 0.01 M monopotassium phosphate (pH 4.5) delivered via a programmed flow. Fluorometric detection wavelengths were 237 nm (excitation) and 550 nm (emission).

### Statistical analysis

All data are expressed as the mean ± SD. Differences between the mean values of multiple groups were analyzed by one-way analysis of variance (ANOVA) followed by Tukey’s post hoc test. The differences between two groups were analyzed using a two-sided Student’s *t* test. Statistical significance was considered at P < 0.05.

## Results

### Effect of scutellarin on DOX-induced changes of MDA levels and LDH activity

Rats treated with a single dose of DOX (20 mg/kg) showed significantly elevated plasma LDH activity (4057.8 ± 107.20 vs. 2032.7 ± 70.95, P < 0.01) and MDA levels (2.083 ± 0.10 vs. 1.103 ± 0.09, P < 0.01) compared with those of the control group (Table 1). The co-administration of scutellarin with DOX resulted in a significant decrease in LDH activity (2595.9 ± 72.73, P < 0.01) and MDA levels (1.380 ± 0.06, P < 0.01) compared with those of the DOX group.

**Table 1:** Effect of scutellarin on DOX-induced changes in MDA levels and LDH activity

Group	MDA (n moles/mg protein)	LDH (U/L)
Control	1.103 ± 0.09	2032.7 ± 70.95
DOX	2.083 ± 0.10**	4057.8 ± 107.2**
DOX + SCU	1.380 ± 0.06*	2595.9 ± 72.73*
SCU	1.126 ± 0.12	2054.3 ± 40.4

Values are expressed as the mean ± SD (n = 8)

* Compared with the DOX-treated group, P < 0.01

** Compared with the control group, P < 0.01

### Effects of scutellarin on the serum concentration of cTnT

**Fig. 1:**
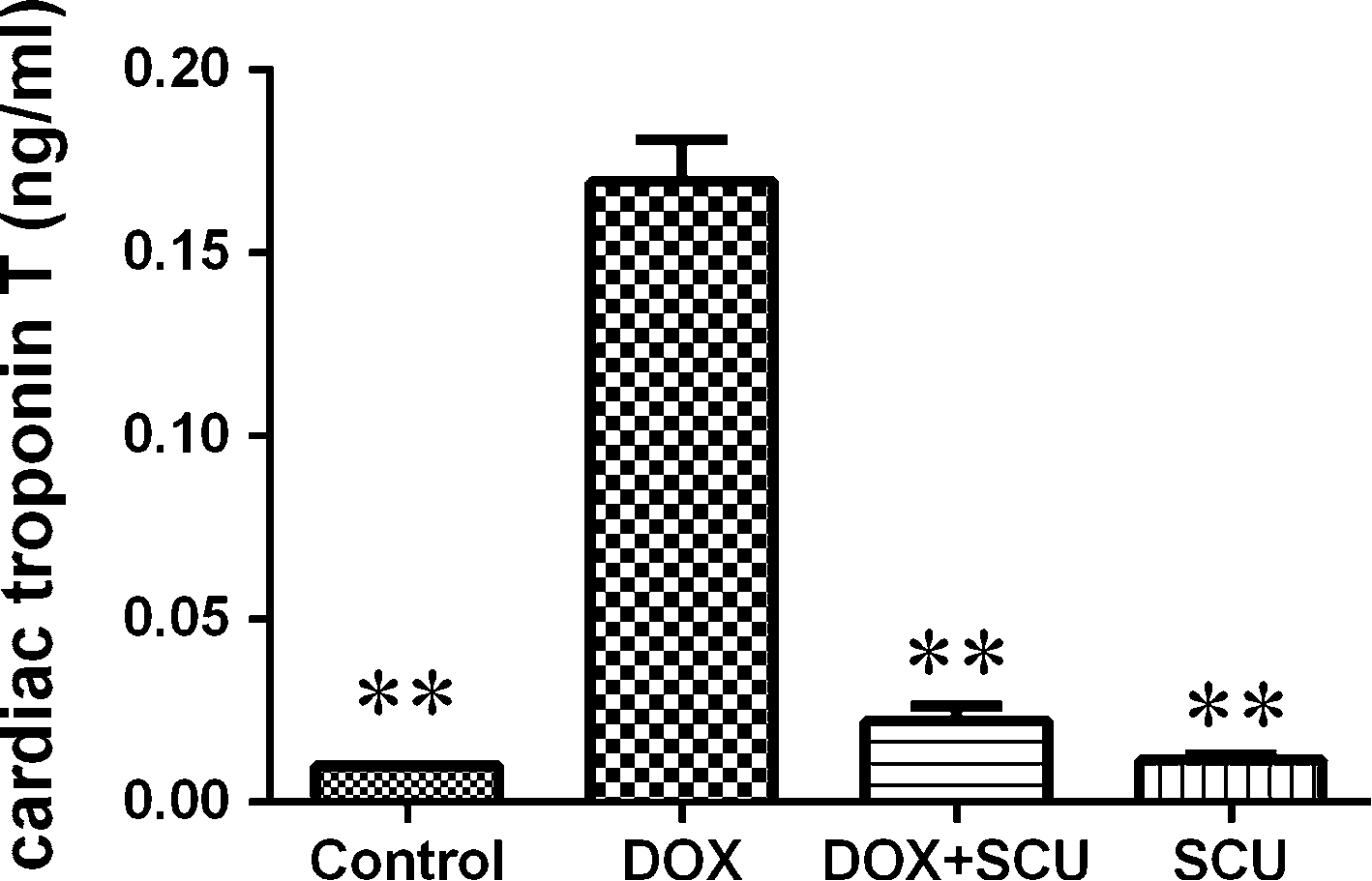
Effect of DOX and/or scutellarin on the concentration of cTn T in serum. **Compared with DOX-treated group, P < 0.01

Cardiac troponins are regulatory proteins of the thin actin filaments of cardiac muscle. cTnT is a highly sensitive and specific marker of myocardial injury. In this study, the cTnT concentration was below the level of detection in the control and SCU groups (<0.01 ng/mL), indicating there was no myocardial injury (Fig. 1). The results showed that 3 days after DOX administration, the average cTnT concentration in the serum of the DOX group was 0.1695 ± 0.0114 ng/mL, which was significantly elevated compared with the control group (P < 0.01). However, upon co-administration with scutellarin for 3 days, the concentration of cTnT in the DOX + SCU group decreased to 0.0222 ± 0.0041 ng/mL (compared with the DOX group, P < 0.01).

### Echocardiographic assessment of cardiac physiological alterations

**Fig. 2:**
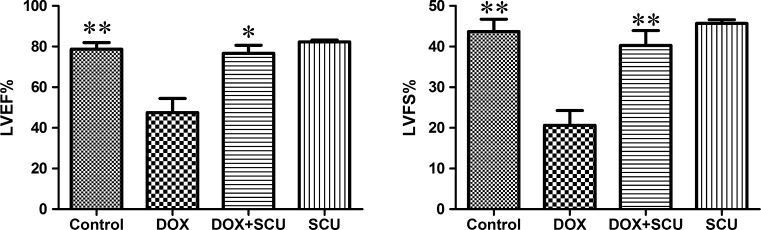
Effects of DOX alone or combined with scutellarin on LVEF and LVFS of rats. *Compared with DOX-treated group, P < 0.05; **compared with DOX-treated group, P < 0.01

Left ventricular function was similar between the control group and the SCU group (Fig. 2). At day 3, significant compromises in LVEF were observed in the DOX-treated group compared with the control group (47.75 ± 15.79 vs. 78.72 ± 7.25, P < 0.01). The LVEF of the DOX + SCU group was close to the level of the control group and higher than that of the DOX-treated group (76.70 ± 3.91, P < 0.05). The LVFS was also significantly lower in the DOX-treated group than that in the control group (20.66 ± 8.06 vs. 43.7 ± 6.76, P < 0.01). The LVFS was improved in the DOX + SCU group compared with that of the DOX-treated group (40.28 ± 3.68, P < 0.01).

### Morphological changes of the myocardium

**Fig. 3:**
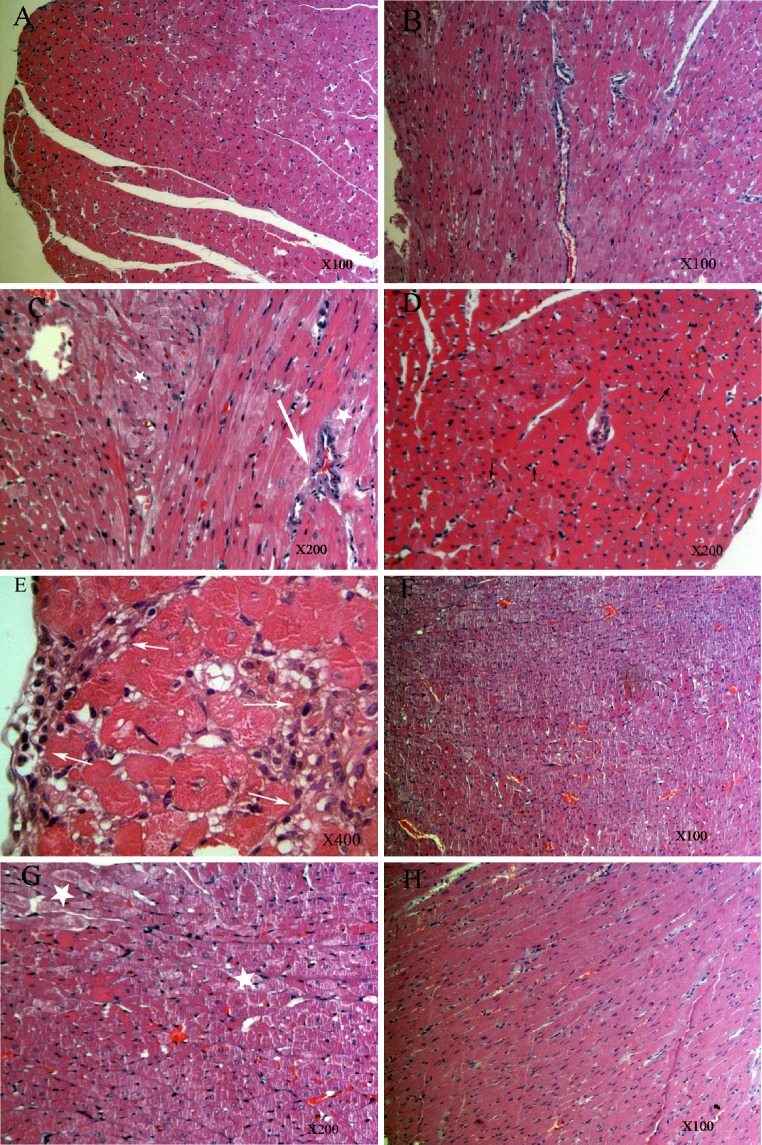
Histopathological examination of rat heart (H&E). Control group (**a**) and SCU-treated group (**h**) showed normal morphology. DOX-treated group shows myocardial fibrosis, myocardial degeneration and necrosis, inflammatory cell infiltration and occasional vacuolization (**b**–**e**). However, only occasional myocardial degeneration and inflammatory cell infiltration was detected in DOX + SCU group (**f** and **g**). (*white arrow* means myocardial fibrosis; *white star* means myocardial degeneration and necrosis, inflammatory cell infiltration; *black arrow* means occasional vacuolization)

In the histopathological examinations, both the control and SCU-treated groups revealed normal heart histology (Fig. 3a, h). The DOX-treated group showed severe cardiac damage. Myocardial fibrosis (indicated by a white arrow), myocardial degeneration and necrosis, inflammatory cell infiltration (indicated by a white star), and occasional vacuolization (indicated by a black arrow) were observed in this group (Fig. 3b–e). However, in the DOX + SCU group, light microscopy investigation revealed slight cardiac damage. Occasional myocardial degeneration and inflammatory cell infiltration were detected, although there was no evidence of fibrosis (Fig. 3f, g).

### Effects of scutellarin on the changes of pharmacokinetic and tissue distribution of DOX

**Fig. 4:**
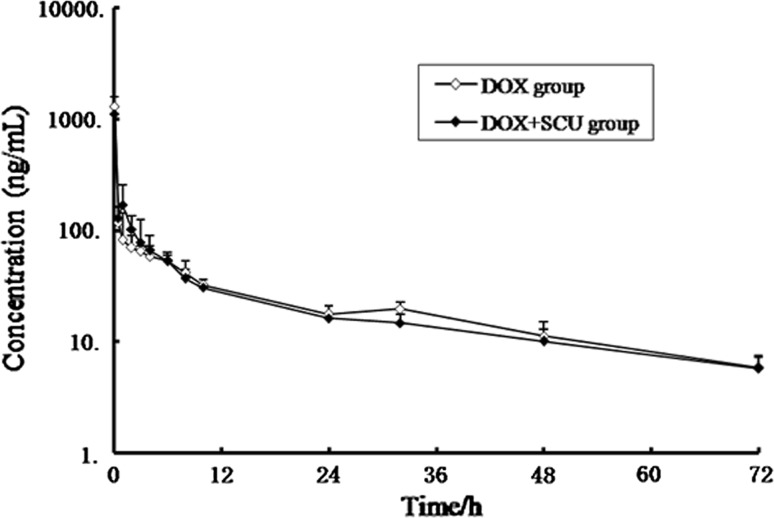
Mean plasma concentration–time curve of DOX along after single dose of 5 mg/kg along or in combination with 5 mg/kg of scutellarin (*n* = 8)

**Fig. 5:**
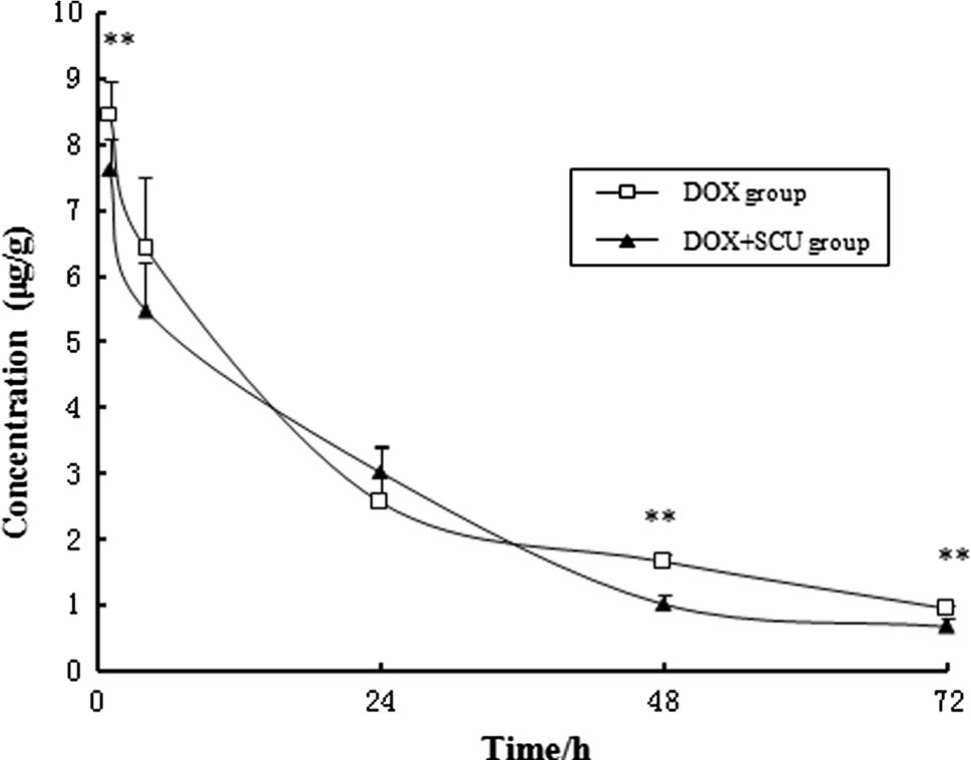
Mean heart concentration–time curve of DOX along after a single dose of 5 mg/kg along or in combination with 5 mg/kg of scutellarin. (*n* = 6). **Compared with DOX-treated group, P < 0.01

HPLC was used to quantify the DOX concentration in plasma and tissues. We studied the effect of scutellarin on DOX pharmacokinetics and tissue distribution within 72 h. The 72 h time-point was selected based on data from pharmacokinetic studies showing that the average terminal half-life of DOX was between 12 and 48 h and because acute cardiotoxicity appears between 48 and 72 h (Iqbal et al. 2008; Todorova et al. 2010). The mean plasma concentration–time curve of DOX is presented in Fig. 4, and the major pharmacokinetic parameters are summarized in Table 2. There were no significant differences in the main pharmacokinetic parameters between the DOX-treated group and the DOX + SCU group. The concentrations of DOX in primary tissues in the tissue distribution are summarized in Table 3. Excluding the heart, there were no significant differences between the two groups for most tissues. The DOX concentration in the heart of the DOX + SCU group was lower than that in the DOX-treated group, and there were significant differences at 1, 48 and 72 h (P < 0.05) (Fig. 5). These results show that scutellarin can reduce heart tissue exposure to DOX without changing the AUC of plasma.

**Table 2:** Major pharmacokinetic parameters of single i.v dose of 5 mg/kg DOX alone or in combination with 5 mg/kg of scutellarin (*n* = 8)

Parameter	DOX group	DOX + SCU group
AUC(0–t)/ng/L*h	1871.42 ± 261.07	1839.58 ± 281.93
AUC(0–∞)/ng/L*h	2100.16 ± 389.68	2028.48 ± 374.27
Cmax/ng/L	1244.64 ± 271.36	1118.63 ± 154.74
Tmax/h	0.0833	0.0833
t1/2z/h	25.36 ± 7.61	24.12 ± 6.67
CLz/L/h/kg	1465.76 ± 271.47	1514.50 ± 258.69

**Table 3:** The concentrations of DOX in tissues after a single dose of 5 mg/kg alone or in combination with 5 mg/kg of scutellarin (*n* = 6)

Group	Blood (ng/ml)	Heart (μg/g)	Liver (μg/g)	Spleen (μg/g)	Lung (μg/g)	Kidney (μg/g)	Muscle (μg/g)	Gut (μg/g)
1 h								
DOX	40.98 ± 3.12	8.47 ± 0.50	7.12 ± 0.84	9.73 ± 1.24	12.70 ± 1.77	14.84 ± 1.64	3.01 ± 0.09	5.22 ± 0.30
DOX + SCU	41.37 ± 2.79	7.63 ± 0.46**	5.60 ± 0.69	8.05 ± 0.20	14.06 ± 1.18	13.24 ± 1.40	2.84 ± 0.50	4.91 ± 0.54
4 h								
DOX	36.89 ± 8.66	6.43 ± 1.07	4.24 ± 0.79	11.99 ± 2.31	9.68 ± 1.89	10.86 ± 1.81	3.59 ± 0.41	4.68 ± 1.07
DOX + SCU	21.24 ± 1.97	5.48 ± 0.72	2.69 ± 0.43	9.33 ± 1.21	9.34 ± 0.97	9.62 ± 0.87	3.34 ± 0.67	4.25 ± 0.55
24 h								
DOX	16.61 ± 2.42	2.57 ± 0.82	1.15 ± 0.23	8.69 ± 0.94	6.44 ± 1.59	5.70 ± 0.64	2.50 ± 0.72	3.48 ± 0.59
DOX + SCU	14.40 ± 3.54	3.04 ± 0.39	1.22 ± 0.33	8.34 ± 1.69	7.16 ± 0.31	5.72 ± 0.60	2.20 ± 0.25	3.63 ± 0.54
48 h								
DOX	6.93 ± 1.13	1.67 ± 0.10	1.11 ± 0.13	10.24 ± 0.58	5.29 ± 0.33	3.03 ± 0.18	0.99 ± 0.09	2.48 ± 0.20
DOX + SCU	5.99 ± 1.46	1.03 ± 0.12**	0.87 ± 0.14	9.68 ± 0.87	4.12 ± 0.27	2.61 ± 0.41	0.89 ± 0.12	1.76 ± 0.28**
72 h								
DOX	4.22 ± 0.54	0.94 ± 0.07	0.76 ± 0.06	9.79 ± 0.83	4.08 ± 0.30	1.91 ± 0.16	0.75 ± 0.04	1.29 ± 0.15
DOX + SCU	3.27 ± 0.89	0.69 ± 0.12**	0.68 ± 0.13	8.57 ± 1.43	3.63 ± 0.64	1.74 ± 0.35	0.65 ± 0.10	1.21 ± 0.11

Values are expressed as the mean ± S.D. (n = 6)

** Compared with the DOX-treated group, P < 0.01

## Discussion

Dose-dependent cardiotoxicity and congestive heart failure remain major limitations in standard and high dose anthracycline chemotherapy, strongly affecting the survival of cancer patients. DOX-induced cardiotoxicity in patients has been subdivided into acute and chronic forms, depending on the occurrence following administration (Outomuro et al. 2007). The cause of DOX-induced acute cardiotoxicity appears to be multifactorial, although most studies support the theory that enhanced oxidative stress and antioxidant deficits play significant roles in DOX-induced acute cardiomyopathy and congestive heart failure (Zhou et al. 2001; Takemura and Fujiwara 2007). Furthermore, cardiocytes have poor antioxidant defense systems compared with other normal tissues. DOX could disturb the antioxidant defense systems and repair pathways (Nikitovic et al. 2014), aggravating the oxidative damage to the heart. Many antioxidants, such as α-tocophenol, ascorbic acid, reduced glutathione, probucol, carvedilol, and others, can protect against DOX-induced acute cardiotoxicity (Yoda et al. 1986; Geetha et al. 1990; Shimpo et al. 1991; Santos et al. 2002; El-Demerdash et al. 2003), although their efficacies are different and clinical results have been disappointing.

Currently, much attention has been paid to the use of phytochemicals as a protective strategy against DOX-induced cardiotoxicity. Flavonoids are a group of polyphenol compounds that exhibit numerous pharmacological properties that are beneficial for human health (Havsteen 2002). Partly due to their radical-scavenging, antioxidative, and iron-chelating properties, flavonoids are considered potential protectors against cardiotoxicity caused by DOX (Kaiserova et al. 2007) or could improve the therapeutic index of DOX (Du et al. 2009).These flavonoids remain in the experimental stage because of their low solubility and oral bioavailability, however, scutellarin widely used for clinical treatment of cerebral insufficiency and peripheral circulation problems in China.

Scutellarin is a polyphenolic flavonoid compound and shows high free radical-scavenging activity, is a highly effective antioxidant and protects cells from oxidative stress. The present study demonstrated that scutellarin could protect against DOX-induced cardiotoxicity and oxidative injury. Rats treated with DOX showed cardiotoxicity as indicated by elevated LDH activity, tissue MDA level and cTnT concentration, and decreased LVEF and LVFS. Cotreatment with scutellarin significantly decreased the LDH activity, MDA level and cTnT concentration, and increased LVEF and LVFS. Histopathological studies showed disruption of cardiac tissues in the DOX groups. Cotreatment with scutellarin could reverse the damage to the cardiac tissues. These results demonstrate that scutllarin’s antioxidative effect may underlie the protection against DOX cardiotoxicity.

Another important factor that could mediate cardiotoxicity is high affinity binding of DOX to cardiolipin, an anionic phospholipid in the inner mitochondrial membrane, which has been proposed as a privileged target (Moulin et al. 2015). The cardiotoxicity of DOX is related to the dose intensity and, to a certain extent, to the peak and cumulative concentrations in heart tissue. Studies in animals and human clinical trials have demonstrated that the tissue distribution of DOX is known to be schedule-dependent. The reduced cardiotoxicity of conventional or pegylated liposome encapsulated DOX is related to the decreased concentration of DOX in the heart.

In the pharmacokinetic and tissue distribution study, scutellarin significantly reduced the concentration of DOX in heart tissues. However, scutellarin did not change the plasma pharmacokinetic parameters, such as the AUC, C_max_, and T_1/2_. Flavonoids are the substrates of some transporters and metabolic enzymes, and a number of studies have investigated the effect of flavonoids on the transport of DOX in tumors and on the metabolism of DOX. These studies indicated that flavonoid-P-gp or MRPs could occur in vivo, resulting in pharmacokinetic interactions (Morris and Zhang 2006). Luteolin and quercetin were reported to regulate transporters, sensitizing tumor cells to DOX (Du et al. 2008, 2009). The cardiotoxicity of anthracyclines has been attributed to the intramyocardial formation of C-13 alcohol metabolites (Mordente et al. 2003). Quercetin has better cardioprotective effects against DOX cardiotoxicity, and it could significantly inhibit the metabolic conversion of DOX to doxorubicinol (Vaclavikova et al. 2008). The aldo–keto reductase inhibitors phenobarbital and rutin also could alter the metabolism, pharmacokinetics, and toxicity of anthracycline (Behnia and Boroujerdi 1999; Kang and Weiss 2003). However, it has not been reported that flavonoids can diminish heart exposure to DOX. Working et al. (1999) reported that DOX-mediated cardiotoxicity is most directly related to the peak plasma and/or tissue concentrations of the drug, although antitumor activity is more closely related to the plasma AUC. According to this conclusion, it is beneficial for cardioprotection that scutellarin reduce DOX accumulation in the heart. Otherwise, the antitumor activity might not be reduced because the AUC of plasma does not change. Recently, Todorova et al. found that glutamine supplementation resulted in a significant reduction of DOX concentration in heart tissues, without a significant effect on tumor DOX concentration (Todorova et al. 2010), although this study did not elucidate the mechanism. The reason for reduced accumulation of DOX in the heart might be that scutellarin competes with a specific transport protein characterized by saturable uptake or active specific efflux-protein, such as multidrug resistance-associated protein (MRP) 2 and organic anion-transporting polypeptide (OATP) 2B1 (Couture et al. 2006; Gao et al. 2012), promoting DOX efflux in the heart. In addition, disruption of DOX-cardiolipin complexes might also result in decreased concentrations of DOX in the heart.

Our study demonstrates for the first time that scutellarin can protect against DOX-induced acute cardiotoxicity through its antioxidant activity and can influence heart accumulation. Scutellarin decreases DOX accumulation in the heart without changing the pharmacokinetics of plasma. Further studies are needed to elucidate the molecular mechanism of scutellarin on cardioprotection of DOX within the heart.
